# The same, only different: what can responses to music in autism tell us about the nature of musical emotions?

**DOI:** 10.3389/fpsyg.2013.00156

**Published:** 2013-04-04

**Authors:** Rory Allen, Reubs Walsh, Nick Zangwill

**Affiliations:** ^1^Department of Psychology, Goldsmiths, University of LondonLondon, UK; ^2^Department of Physiology, Anatomy and Genetics, University of OxfordOxford, UK; ^3^Department of Philosophy, University of DurhamDurham, UK

We propose addressing the theme of this special issue by examining the affective responses that music evokes in the individual. The logical first step is to enquire how far these responses resemble naturalistic emotions, i.e., those that are not specifically musical, but have ordinary non-musical content. The literature is ambivalent on this. Many authors suggest that whilst certain emotions are exclusive to music (Scherer and Zentner, 2008), there is considerable overlap between “musical” and “naturalistic” emotions (Zentner et al., 2008); others deny that musically induced emotions are naturalistic (Konecni, 2005, 2008), a view elaborated by the nineteenth century critic Eduard Hanslick (Hanslick, 1986; see also Kivy, 2001, 2009; Zangwill, 2004, 2007, 2011).

Perhaps consideration of music's origins might clarify the issue. If the universality of music in human society were the consequence of biological selection (Huron, 2001; Mithen, 2009), this would support the naturalistic interpretation. If music is, literally, in our DNA, then human responses to music will form part of the normal repertoire of emotions. However, Patel (2008) has given reasons for rejecting the evolutionary theory in favor of the idea of music as “transformative technology,” implying that it is the outcome of cultural, not biological evolution. An important recent study has provided perhaps the first experimental evidence for this. Inspired by cultural transmission theory (Boyd and Richerson, 1985), MacCallum et al. (2012) demonstrated the effectiveness of consumer selection in generating music out of noise in a Darwinian model of cultural evolution. This suggests that music has evolved to satisfy human aesthetic criteria, not vice versa.

Moreover, the universality of music goes hand in hand with an extraordinary diversity, as MacCallum et al. point out (the need to explain this being one of the drivers for their study). Language's acknowledged genetic basis is associated with deep structural similarities between human languages. Yet the “languages” of music have little in common cross-culturally. Javanese gamelan music uses two scales, both totally different from the 12 note Western scale (Patel, 2008, p. 19). West African drum music employs a feature, the “time line,” unknown to Western tradition (Agawu, 2006) and a rhythmic structure which has “a richness and subtlety found in no other music” (Temperley, 2000, p. 79). Japanese hogaku is “a music erected upon such a different foundation and animated by so different an aesthetic” that it has essentially nothing in common with the Western classical corpus (Dean, 1985, p. 147).

Taking the longitudinal view, and focusing on just one musical sub-culture, that of Western music, its development during the past millenium from Gregorian plainchant to modern electronic music illustrates that the evolution of music operates several orders of magnitude faster than human evolution. Languages, by comparison, show continuities in deep structure that can be traced back 10,000 years and even beyond (Dunn et al., 2005). The conclusion seems inescapable. The evolutionary boot is on the other foot: it is music that has evolved to fit humans, not vice versa. But if human responses to music are not the result of biological evolution, the *a priori* argument that these affective responses must correspond to naturalistic emotions falls away.

On the other hand, Patel's model not only explains the speed and diversity of musical development, but also suggests an answer to our initial question. Borrowing Patel's analogy, humans migrating out of Africa into Europe would have found the warmth of fire a life-giving substitute for the warmth of tropical sunshine, and the fact that it was not in all respects identical with sunshine did not detract from its value. For some purposes the warmth of fire would have proved superior: it does not induce sunburn, and one can cook with it. Similarly, the emotional warmth induced by music need not be identical with that provided by any natural emotion, and in some respects the differences may enhance its value: what we call the “sadness” in music may strike us as so pleasant partly because it does *not* induce real sadness in listeners.

The outcome of the study reported in Allen et al. (2013), though not designed with this purpose, turns out to have a bearing on the question. Matched adult autism and control groups were compared on the autonomic and cognitive components of their emotional responses to a standard list of music items (Quintin et al., 2011). Whereas the groups responded similarly at an autonomic level, they differed at a cognitive level precisely as would be expected if autonomic arousal causes cognitive arousal. Regression analysis suggested that the causal chain was mediated by levels of type II alexithymia, or the cognitive inability to interpret and verbalize the lower level autonomic or visceral aspects of emotion (this has high comorbidity with autism: Berthoz and Hill, 2005). The mediation interpretation was robust: levels of alexithymia, and verbalization of emotion, also correlated significantly within the control group.

How should we interpret this result? If we assume as a working hypothesis that affective responses to music are naturalistic, it follows that they should be activated via one or both of the two principal routes to emotion induction, the “fast” (thalamic) and “slow” (cortical) routes (LeDoux, 2000). The fast route rapidly alerts the autonomic nervous system, priming the body to take immediate action to cope with a potential emergency, or opportunity; only subsequently are the higher cognitive functions recruited, to verify, elaborate, and possibly revoke the fast track responses to an alert. With the slow route, incoming sensory signals are first appraised by the higher centers of the brain; if found emotionally relevant, they induce autonomic and bodily arousal. In both cases, however, for the arousal to be considered a naturalistic emotion, autonomic and cognitive components must both eventually be activated, and should be congruent with one another.

The results from Allen et al. (2013), in particular the mediation analysis, appeared inconsistent with a naturalistic slow track route for the induction of musical emotions in our study. It might be argued that this was because the individual musical extracts were short (30 s): we have no difficulty with accepting that higher level cognitive processes, including such mechanisms such as the ITPRA sequence described by Huron (2006), are important for the aesthetic aspects of musical appreciation in extended listening, though many cognitive effects may occur in as brief a period as three seconds (Plazak and Huron, 2011). The pleasure induced by music activates normal dopamine reward and anticipation circuits (Salimpoor et al., 2011). However, this is irrelevant since pleasure in general, and aesthetic pleasure in particular, is not an emotion.

Juslin and Västfjäll (2008) propose six mechanisms for emotion induction by music (they exclude cognitive appraisal from the outset, we think correctly). These are brain stem reflexes, evaluative conditioning, emotional contagion, visual imagery, episodic memory, and musical expectancy. Of these, the first and last have been discussed above (in the “fast route,” and the ITPRA mechanisms respectively). Emotional contagion involves no appraisal process and we would include it in our fast route mechanism. Evaluative conditioning and episodic memory both rely on arbitrary associations, essentially independent of any properties of the music. In the case of visual imagery, Juslin and Västfjäll cite no clear experimental evidence of any consistent causal link between musical structure and particular visual images, let alone between music and any emotions induced through that mechanism.

We conclude that musical emotions, if they are emotions at all in the conventional sense, are fast track emotions. With naturalistic fast track emotions, the autonomic arousal component should be complemented by the appropriate cognitive counterpart. According to Huron (2011), this is not the case, at least for negative autonomic responses such as sadness. Huron considers that the autonomic system responds automatically to such music with a kind of “sham pain,” but we enjoy “sad” music because the conscious brain realizes that the situation is not threatening, and responds with relief, aided by the liberation of prolactin, so that the net effect is pleasurable. If we accept Huron's ITPRA model, we may plausibly speculate that a further effect might be due to the combination in music of a high degree of order and pattern, and sufficient variety to make it unpredictable. This acts as intellectual catnip to the pattern detection and prediction aspects of executive functioning. The analysis of these patterns may be sufficiently interesting to, and demanding of, the higher brain centers that they are distracted from their normal role in monitoring autonomic arousal, thus permitting the arousal induced by the fast track mechanism to persist in defiance of its lack of congruence with reality. These processes would enable the generation of patchwork emotion states comprising activation of combinations of different brain circuits not found in naturalistic emotions. We might call these states “chimerical,” after the composite lion/goat/serpent creature of Greek myth. They would be sufficiently rewarding to make us wish to repeat the listening experience, and this could be a driver, in a model such as that of MacCallum et al. (2012), for music to evolve ways of generating ever more desirable chimerical combinations.

Two questions suggest themselves. Firstly, if musical emotions are indeed not naturalistic emotions, how can we account for the stubbornly persistent illusion that they are? Secondly, how is it that music has the ability to induce such powerful affective states, if indeed they are unnatural? On the first question, we have long known that experiencing the physiological counterpart of an emotion can lead to the brain's attributing the state to a naturalistic emotion even when the cognitive counterpart is not present (Schachter and Singer, 1962). We suggest that this kind of unconscious confabulation may be happening when a listener is asked to describe their emotional experiences, especially if there is social pressure to feel the “appropriate” emotions. Moreover, as pointed out in Zentner et al. (2008), p. 494, some experimental protocols embody a theory of emotion developed outside music that compels the use of standard emotion words.

As to the second question, Juslin (2000, 2001) has argued that musical instruments act as “superexpressive voices,” which enhance and exaggerate the emotionally expressive components of the human voice. This theory is a perfect counterpart, for music, to that of Ramachandran and Hirstein (1999) in the visual arts, with their notion of “supernormal stimuli” (though we should note that due priority should be accorded to Aldous Huxley: see his 1956 book “Heaven and Hell”; also Allen and Heaton, 2010, p. 255). However, composers have an important advantage over visual artists, who cannot precisely control how viewers scan an artwork, whereas listeners cannot avoid hearing the notes in the intended order: this allows for all the sophisticated mechanisms for generating tension and satisfying, or violating expectation as described in Huron's ITPRA model (Huron, 2006).

Concluding on a constructive note, though affective responses to music may lack validity as naturalistic emotions, they are not for that reason valueless. Music can undoubtedly influence mood, indeed we know that mood management is one of the main reasons people give for listening to music (North et al., 2004). It is plausible that music has the ability to vary mood states in a positive way along both axes of the two-dimensional arousal space described by Thayer (1978), leading to satisfying alterations of mood from tense to calm, and from dull to excited. Such uses were clearly described by participants with autism in Allen et al. (2009): see also Bhatara et al. (2010). Incidentally, the lack of sophisticated emotion descriptors cited in Allen et al. (2009) suggests, in the light of the present paper, that our participants were actually *more* insightful into the true nature of their affective responses to music than typically developing individuals, a nice reversal of the usual representation of autism as a syndrome of deficits.

It was proposed in Allen and Heaton (2010) that the apparent preservation of affective responses to music in neurodevelopmental disorders such as autism, might be used as a means to repair the link between autonomic and cognitive components of emotion where this link is damaged or underdeveloped. The suggestion originated from the personal experience of the second author who had found that autism, with its associated difficulties in learning about emotions via the usual route of social interaction, did not prevent the induction by music of intense affective states, in an unthreatening context, which led to a better understanding of naturalistic emotions. A pilot study under the auspices of the Baily Thomas Charitable Fund currently being conducted by the first author is exploring whether associative learning can be used to help people with type II alexithymia by teaching them, via musical extracts, to attach cognitive labels to their autonomic arousal states. Very preliminary results suggest that our procedure does produce measurable benefits (pending formal publication, some further details of the study can be found online: Allen et al., 2012). Musical emotions possess some of the characteristics of naturalistic emotions and lack others, and we suggest that it is this dual nature which may make them useful in treating conditions where emotional processing is partially preserved, and partially disrupted. If this viewpoint is correct, then their value in this context is precisely because, like individuals with autism, they are both the same and different.
